# Fabrication, Modeling and Characterization of Multi-Crosslinked Methacrylate Copolymeric Nanoparticles for Oral Drug Delivery

**DOI:** 10.3390/ijms12096194

**Published:** 2011-09-23

**Authors:** Ndidi C. Ngwuluka, Viness Pillay, Yahya E. Choonara, Girish Modi, Dinesh Naidoo, Lisa C. du Toit, Pradeep Kumar, Valence M.K. Ndesendo, Riaz A. Khan

**Affiliations:** 1 Department of Pharmacy and Pharmacology, University of the Witwatersrand, 7 York Road, Parktown, 2193, Johannesburg, South Africa; E-Mails: Ndidi.Ngwuluka@students.wits.ac.za (N.C.N.); Yahya.Choonara@wits.ac.za (Y.E.C.); Lisa.DuToit@wits.ac.za (L.C.T.); Pradeep.Kumar@students.wits.ac.za (P.K.); Valence.Ndesendo@wits.ac.za (V.M.K.N.); 2 Division of Neurosciences, Department of Neurology, University of the Witwatersrand, Johannesburg, 2193, South Africa; E-Mail: gmodicns@mweb.co.za; 3 Division of Neurosciences, Department of Neurosurgery, University of Witwatersrand, Johannesburg, 2193, South Africa; E-Mail: dineshnaidoo@yahoo.com; 4 Department of Medicinal Chemistry, College of Pharmacy, Qassim University, Qassim 51452, Saudi Arabia; E-Mail: kahnriaz@gmail.com

**Keywords:** nanotechnology, nanoparticles, nanocapsules, methacrylate copolymer, chitosan, oral drug delivery, bioavailability, crosslinking, molecular mechanics simulations

## Abstract

Nanotechnology remains the field to explore in the quest to enhance therapeutic efficacies of existing drugs. Fabrication of a methacrylate copolymer-lipid nanoparticulate (MCN) system was explored in this study for oral drug delivery of levodopa. The nanoparticles were fabricated employing multicrosslinking technology and characterized for particle size, zeta potential, morphology, structural modification, drug entrapment efficiency and *in vitro* drug release. Chemometric Computational (CC) modeling was conducted to deduce the mechanism of nanoparticle synthesis as well as to corroborate the experimental findings. The CC modeling deduced that the nanoparticles synthesis may have followed the mixed triangular formations or the mixed patterns. They were found to be hollow nanocapsules with a size ranging from 152 nm (methacrylate copolymer) to 321 nm (methacrylate copolymer blend) and a zeta potential range of 15.8–43.3 mV. The nanoparticles were directly compressible and it was found that the desired rate of drug release could be achieved by formulating the nanoparticles as a nanosuspension, and then directly compressing them into tablet matrices or incorporating the nanoparticles directly into polymer tablet matrices. However, sustained release of MCNs was achieved only when it was incorporated into a polymer matrix. The experimental results were well corroborated by the CC modeling. The developed technology may be potentially useful for the fabrication of multi-crosslinked polymer blend nanoparticles for oral drug delivery.

## 1. Introduction

Despite the challenges posed by the oral route on certain drugs such as Narrow Absorption Window drugs and alternative routes being employed, the convenience of the oral route impels the quest to enhance peroral delivery of drugs. Poor or suboptimal therapeutic efficacy is usually obtained from drugs due to poor oral bioavailability which could be caused by biopharmaceutical, physiological or drug inherent factors. The two major factors affecting bioavailability are absorption and metabolism [1], while other sub-factors relating to these two major factors are the physicochemical, biopharmaceutical and physiological factors such as solubility, permeability, particle size, chemical nature of the drug, enzymes, membrane transporters, effects of foods, gastrointestinal transit time, pH as well as the type and process of the formulation [2–4]. Technologies that have been employed to improve oral bioavailability include micronization [5,6], prodrugs [7–9], salt formation [10], absorption enhancement [11], wetting (with a wetting agent) and spray drying [12], solid dispersions [13,14], micellation [15], emulsification [16,17], micro- and nano-emulsification [18–21], self-emulsification [22,23], utilization of cyclodextrins [24,25], chemical modification [26,27], sustained drug delivery, gastroretention and more recently, nanotechnology [28,29].

Nanotechnology is the study, fabrication, characterization and application of structures and materials at the nanometer scale. Pharmaceutical Nanotechnology employs mainly polymers to design, fabricate, characterize and apply nano-carriers for therapeutic purposes. While the field of engineering may describe nanoparticles as particles within 0.1–100 nm, nano-drug carriers are less than 1 μ [30,31], which is mainly due to the fact that they are fabricated with macromolecules, natural and synthetic polymers, lipids and proteins. Nanostructures that have been employed for drug delivery include polymeric nanoparticles, liposomes, dendrimers, nanoemulsion, solid lipid nanoparticles, nanocapsules and polymeric nanomicelles [32–39].

Nanotechnology has been employed to improve bioavailability of certain drugs where the difference when compared to the conventional system has been found to be significant. An *in vivo* study of curcumin-loaded nanoparticles was found to have increased in bioavailability by at least nine-fold compared to curcumin administered with piperine as an absorption enhancer [40]. Other drugs with oral bioavailability enhanced by nanotechnology include cyclosporin-A [41], amphotericin B [42], insulin [43,44], HIV-1 protease inhibitors [45], elcatonin [46], paclitaxel [47], estradiol [48], salmon calcitonin [49], camptothecin [50] and mifepristone [51].

Nanostructures are fabricated utilizing a combination of technologies which include polymerization [52,53], emulsion solvent evaporation [54], salting out, emulsification-diffusion and nanoprecipitation [55–57]; supercritical fluid technology [58,59], coacervation and ionic gelation/crosslinking [60–62]. Crosslinking is a process of introducing bonds which may be chemical (covalent bonds) or physical (ionic bonds) between chains of the same material or different materials. It is basically an interaction that joins two molecular units thereby altering the material for characterization and improved functional properties.

The aim of this study therefore, was to explore the feasibility of preparing levodopa-loaded nanoparticles from a methacrylate copolymer/methacrylate copolymer blend and explore various approaches of achieving sustained drug release from the nanoparticles. The method employed involved the application of miscible polymers in interaction with a phospholipid as the lipid component and a crosslinking agent with subsequent addition of a sequestrator as another crosslinking agent in a multicrosslinking technology to fabricate polymer-lipid (poly-lipo) nanoparticles. The nanoparticles were characterized by employing techniques such as: (i) size and surface analysis; (ii) morphology analysis through digital microscopy, transmission electron microscopy (TEM) and scanning electron microscopy (SEM); (iii) FTIR spectroscopy; (iv) *in vitro* drug release studies; and v) magnetic resonance imaging. To mechanistically elucidate the crosslinking mechanism of lecithin and sodium tripolyphosphate with respect to polymethacrylate (Eudragit^®^)/chitosan and chitosan, respectively, we employed computer-aided modeling of the three-dimensional structure of the active residues of the crosslinkers with the respective substrate to predict the possible orientation of residues most likely to affect the substrate preference.

## 2. Results and Discussion

### 2.1. Modeling, Changes in pH and Absorbance of Poly-Lipo Nanoparticles during Fabrication

**Figure 1:**
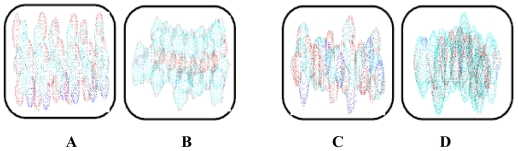
The molecular surface, topology and spaces of the polymers–chitosan and methacrylate copolymer for incoming ligands.

**Figure 2:**
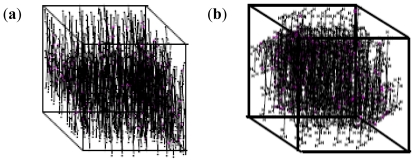
Space-fill/matrix occupation diagram showing maximum occupancy in unit area for (**a**) chitosan; and (**b**) methacrylate copolymer.

**Figure 3:**
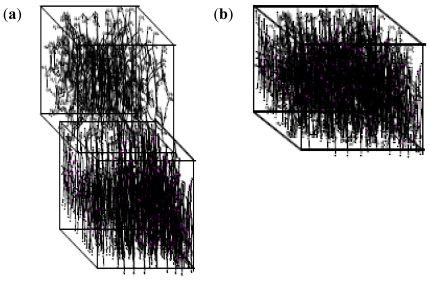
(**a**) Space-fill/matrix occupation diagram showing the maximum occupancy of methacrylate copolymer and chitosan before blending or fusion in a unit area for equal number of molecular weighted polymers (both methacrylate copolymer and chitosan-approx mol weight ~12K amu); and (**b**) fused polymeric matrix containing methacrylate copolymer and chitosan.

**Figure 4:**
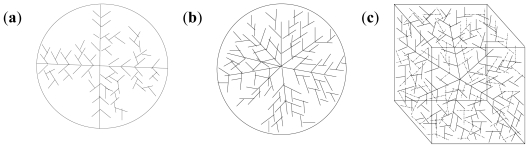
Tree branching pattern: (**a**) octree representation; (**b**) two-dimensional depiction; and (**c**) three-dimensional representation.

Polymeric miscibility was observed between the methacrylate copolymer and chitosan with no visible interactions. Hence, the enhancement of the individual properties of the polymers was envisaged to be through blending. Methacrylate copolymer is not as viscous as chitosan due to the fact that it has a lower molecular area and topology. Hence, its chemical structure possesses more room for incoming entities while chitosan has a larger molecular area with less internal spaces and therefore requires less TPP crosslinking compared to the methacrylate copolymer. The similar molecular weight units (MW-methacrylate copolymer 968 g/mol and chitosan 972 g/mol) for both polymers were energetically minimized qualitatively (as no numerical values of energies were computed by the software) using ACD software in molecular modeling based on three-dimensional (3D) structures of the polymeric units but depicted as dot models in Figure 1a–d:

The models obtained were assessed for the maximum unit area occupation within the given (universally equal) rounded squares defined as unit spaces for the static state of the polymeric units as follows: (A) chitosan (equimolecular weighted units) at the maximum unit area occupation with the highest surface area in a particular 2D dimension; (B) methacrylate copolymer (equimolecular weighted units) at the maximum unit area occupation with highest surface area in 2D dimension of the model; the most area occupancy state; (C) chitosan at the maximum unit area occupation with lowest surface area in 2D dimension of the model at static state; the least area occupancy for the rounded square sizing unit, (D) methacrylate copolymer (equimolecular weighted units) at the maximum unit area occupation with lowest surface area in 2D dimension of the model in static state; the least area occupancy state. Thus, the difference in edges and other 2D matrix areas in the unit space were available to the incoming ligands or low molecular weight (LMW) entities. However, in the 3D unit space, the difference in area was 0. However, for the longer strands of the polymeric molecule, one dimension of the X, Y or Z was comparatively inconsequential or least effective and at a larger scale, the structures followed the 2D modeling for available spaces and facility for entry of ligands/LMW entities. The 3D illustrations of the maximum area of occupancy of chitosan and methacrylate copolymer as well as the fusion of the two polymers are shown in Figures 2 and 3:

The nanoparticle synthesis (with incoming entities-lecithin, levodopa and TPP incorporated into the polymeric matrix) may follow either of seven patterns depending on the space, sizes of particles being formed initially and the presence or absence of turbulence. These patterns are tree branching, nodal space fillings, cone array formations, mixed triangular formations, linear patterns, chaotic patterns and mixed patterns. Figure 4 shows the octree, 2D and 3D representations of the tree branching pattern. The central cross-point (+) in Figure 4a denotes the progenitor point for the nanoparticle synthesis start-up which is followed by distribution to available spaces in an evolving 3D patterning-placement of emerging nanoparticles. The tree formation is dependent on the space available in the 3D matrix area following the branching pattern where the differentiations are outer and inner (back) space bounds depending upon the accommodating space for the particles in all directions.

**Figure 5:**
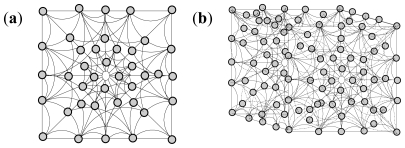
Nodal space fillings: (**a**) two-dimensional; and (**b**) three-dimensional representation.

Nodal space filling pattern (Figure 5) was dependent upon the space-fill in nodal points where the matrix accommodates the emerging nanoparticles while the node generation was dependent upon the changing coordinates of the matrix and their distance parameters (which could accommodate the incoming particles). The nanoparticle emergence in this order of patterning is dependent on the available space, size and distribution of the nanoparticles.

**Figure 6:**
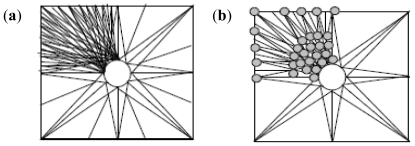
(**a**) Two-dimensional depiction of cone-array formations; and (**b**) particle development, crowding and thinning of the density (space versus number of particles).

The progenitor area for a cone-array pattern is a circle in the middle as shown in Figure 6. The formation of nanoparticles is configured by cone formations where the single lines represent the initial alignment and the merged lines depicting the cone formations onto which nanoparticles align simultaneously as they are formed in the medium. The cone-arrays’ distribution within the matrix is dictated by the physicochemical properties of the polymeric matrix, the formation of the nanoparticles, their optimal formation, grouping and ultimately the thinning density of the emerging nanoparticles which may be influenced by synthesis restrictions, raw materials and space limitations.

**Figure 7:**
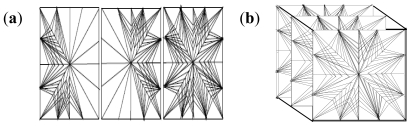
Frontal view of mixed triangle formations of nanoparticles: (**a**) two-dimensional slice representation; and (**b**) three-dimensional slice representation.

The particle formation in the mixed triangle formation leads toward a triangular shape from the progenitor point and the end point tapering in 3D spaces attempting to fill the unit matrix area in all plausible directions as shown in Figure 7. The triangles are formed due to the differentiation of the nanoparticles and changing shape of the matrix under applied force during experimental conditions from nanoparticles which are located along the triangular lines.

**Figure 8:**
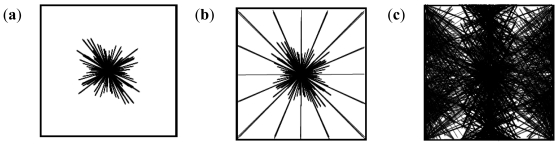
Linear pattern of nanoparticle formation: (**a**) progenesis and initial propagation; (**b**) more lines of propagation; and (**c**) two-dimensional model of the matrix containing nanoparticles on the lines.

Figure 8 represents the linear pattern with progenesis also from the middle with formation of more lines of propagation while the nanoparticles are formed on the lines.

**Figure 9:**
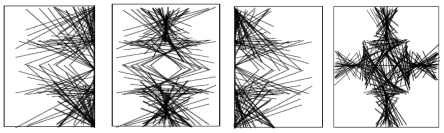
Two-dimensional chaotic pattern of nanoparticle formation.

The chaotic pattern is dependent on the progenesis point and its location in a comparatively non-turbulent medium. The formation of nanoparticles may follow any design in the X, Y, Z, top or bottom arrangement of the matrix (Figure 9).

**Figure 10:**
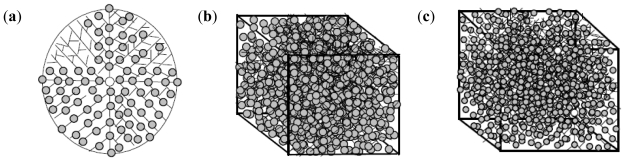
Mixed patterns of nanoparticle formation based on randomizations: (**a**) two-dimensional slice of a single progenitor random patterning; and (**b**) three-dimensional model of single embedded progenitor in a chaotic-random mix state (**c**) three-dimensional model of the multiple progenitors in a chaotic-random mix state based on turbulence in the medium.

The mixed pattern in Figure 10 is based on randomization which can occur with a single progenitor or multiple progenitors. Multiple progenesis is initiated by the presence of turbulence in the medium.

In summary, it is envisaged that the pattern of nanoparticle formation that occurred in the experimental conditions (agitation) employed in this study is either mixed triangle formation or mixed patterns.

Lecithin is an anionic phospholipid and surfactant which physically crosslinks the native cationic methacrylate copolymer and methacrylate copolymer/chitosan polymeric solutions by electrostatic interactions to produce polymer-lipid (poly-lipo) nanoparticles. Studies have confirmed the interactions between chitosan and phospholipids (lecithin) [63–68], while the interaction between methacrylate copolymer and lecithin was observed in this study. The act of sequestration and crosslinking of TPP further binds the components in a nanoparticulate complex. The addition of TPP increased the degree of crosslinking which in turn influenced rate of drug release from the poly-lipo nanoparticles. Increase in concentration of polymers and other excipients increased the pH of the nanosuspensions (Table 1). For the polymethacrylate copolymer/chitosan blend, pH increased with the addition of each component but the increase was more pronounced when TPP was added. However, with methacrylate copolymer alone - B9 and B18, there was no change in pH from the addition of levodopa to that of lecithin.

White methacrylate copolymer nanoparticles and turbid methacrylate copolymer/chitosan were formed in the presence of lecithin and TPP. On addition of lecithin, a color change (colloidal dispersion) was observed indicating the presence of interactions between lecithin (phospholipids) and the polymeric solution. The color change could also be due to the formation of capsular wall and surfactant activity. Furthermore, the color change may be depicting energy perturbation. The oxygen excitation provides the color change–protons are absorbed and the rest of the visible spectrum wavelength is reflected back. The addition of TPP to chitosan or methacrylate copolymer/chitosan blend gave a creamer color because of the oxygen-related functions (excitable oxygen atoms, conjugated oxygen containing groups in higher degree are present in chitosan and TPP). The degree of absorbance of visible light increases as lecithin and TPP are added to polymeric solutions (Table 2) which is also an indication of color change and subsequent interactions between polymeric solution and the ionic agents (lecithin and TPP). However, it is observed that addition of TPP to methacrylate copolymer-lecithin blend led to decrease in absorbance. This is due to the fact that chemical infrastructure of methacrylate copolymer requires a higher quantity of TPP than utilized to achieve sufficient particulate complexation. An increase in TPP generates drier, free flowing particles. This is attributed to increased quantity of TPP causing less room in the particles matrices for solvent and water molecules. From the modeling, the morphology of the nanoparticles formed is suggested to be nanocapsules, loosely filled hollow capsules or solid spherical particles.

### 2.2. Size and Surface Charge Analyses of Poly-Lipo Nanoparticles

The average particle sizes of the nanoparticles after the addition of lecithin ranged from 152 nm for methacrylate copolymer only to 321 nm for methacrylate copolymer/chitosan blend while the zeta potential ranged from 15.8 to 43.3 mV. As the quantity of chitosan increased, the particle size increased. Furthermore, since the degree of crosslinking was increased by the addition of TPP, the particle size increased to average particle size of 424 nm. The polydispersity index ranged from 0.19 to 0.61. Formulation of poly-lipo nanoparticles as nanosuspension may require increased quantity of TPP to ensure free flowing nanoparticles. When administered as nanosuspension, it is envisaged that, based on the mucoadhesive property of chitosan, the nanoparticles will adhere to the mucosal wall of the duodenum while levodopa is being released into the systemic circulation. Hence, the focus may be on obtaining free flowing particles to ensure effective packaging rather than obtaining a particle size below 100 nm.

### 2.3. Fourier Transform Infra-Red (FTIR) Spectroscopy of the Poly-Lipo Nanoparticles

**Figure 11:**
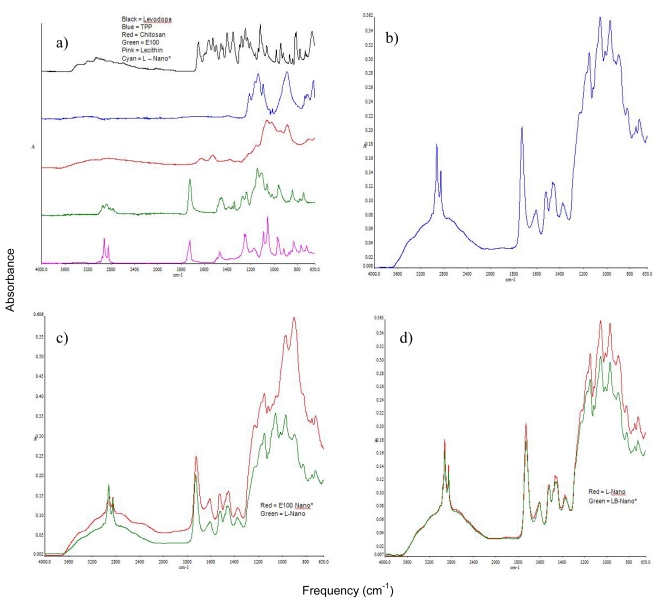
FTIR spectra of: (**a**) the various components employed for fabrication of nanoparticles as well as the fabricated levodopa-loaded nanoparticles; (**b**) levodopa-loaded nanoparticles; (**c**) methacrylate copolymer nanoparticles and methacrylate copolymer/chitosan nanoparticles; and (**d**) levodopa- and levodopa/benserazide-loaded nanoparticles.

The spectra as shown in Figure 11 exhibited the chemical structural transitions that had occurred during nanofabrication by multi-crosslinking. In comparison with the spectra of the native polymers, the spectra of the nanoparticles showed absence of some peaks found in the native polymers such as at frequencies of 2769.74 cm^−1^ and 1268.73 cm^−1^ for methacrylate copolymer; 3357.51 cm^−1^, 1590.66 cm^−1^ and 1024.66 cm^−1^ for chitosan with emergence of new peaks after crosslinking at 1605 cm^−1^ which was found in methacrylate copolymer nanoparticles as well as the blend (methacrylate copolymer/chitosan); 1519 cm^−1^ in methacrylate copolymer which slightly shifted in the blend to 1518.75–1522.24 cm^−1^ (envisaged to be determined by the degree of crosslinking in each nanoparticles formulation). Also, the peaks in the native polymers, which may be considered to still exist, shifted. For example: the peak at 2949.11 cm^−1^ in methacrylate copolymer shifted to 2923.91 cm^−1^; 1722.39 cm^−1^ shifted to 1724.86 cm^−1^ and 891.80 cm^−1^ in chitosan shifted to 889.79 cm^−1^. The distinct aldehyde functional group (C=O) found in methacrylate copolymer at 1722.46 cm^−1^ and in lecithin at 1722.16 cm^−1^ was found in the nanoparticles at 1727.97 cm^−1^ (Figure 11a and b). The disappearance of peaks and emergence of new peaks is attributed to the interactions between the ionic agents leading to polymer-lipid-salt particulate complex. The differences between spectra of methacrylate copolymer nanoparticles and methacrylate copolymer/chitosan nanoparticles as shown in Figure 11c are found at the O-C bands (1056.09 cm^−1^ and 1013.64 cm^−1^) and at 824.73 cm^−1^ of methacrylate copolymer while the other peaks which are similar in both did not absorb at exactly the same place. Some peaks have a difference in absorbance frequencies of 1, 2 or 3 cm^−1^. No additional difference was found when benserazide was incorporated (Figure 11d) which may be an indication that the drugs–levodopa and benserazide did not interact with other components or due to the small quantity of benserazide and its similarity in structure to levodopa; there was no observed distinct peak for it (benserazide).

### 2.4. Surface Morphology of the Poly-Lipo Nanoparticles

**Figure 12:**
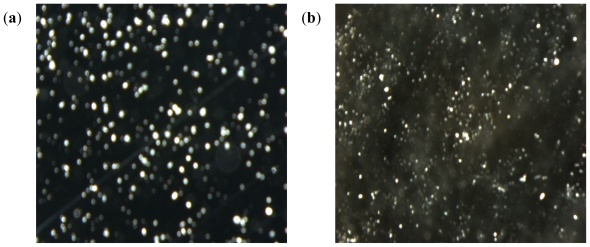
Digital images of: (**a**) methacrylate copolymer/chitosan crosslinked with lecithin alone; and (**b**) mulit-crosslinked methacrylate copolymer nanoparticles (magnification ×32).

Spherically structured nanoparticles were observed as viewed under the digital microscope before lyophilization. Figure 12 shows digital images of methacrylate copolymer/chitosan crosslinked with lecithin only and multi-crosslinked methacrylate copolymer nanoparticles. The smaller sizes of methacrylate copolymer nanoparticles as compared to the polymeric blend with chitosan were further confirmed by the digital images.

**Figure 13:**
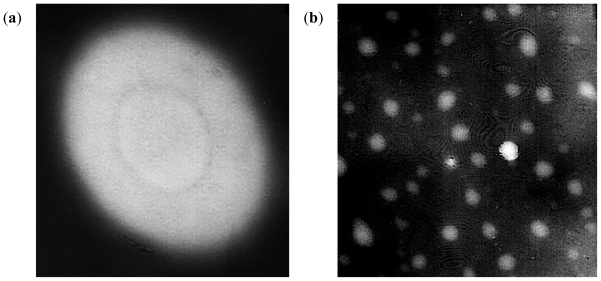
Transmission electrom microscopic images of levodopa-loaded polymethacrylate copolymer/chitosan poly-lipo nanoparticles: (**a**) magnification ×20000; and (**b**) magnification ×8000.

**Figure 14:**
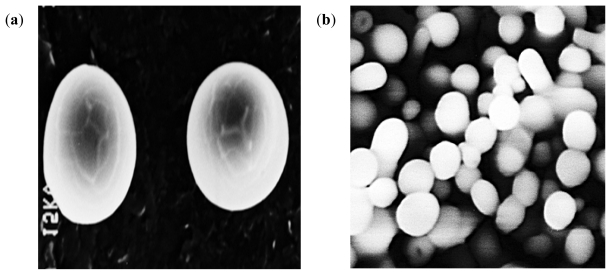
Scanning electron microscopic images of levodopa-loaded polymethacrylate copolymer/chitosan poly-lipo nanoparticles: (**a**) magnification ×5000; and (**b**) magnification ×5500.

TEM confirmed the capsular wall of the levodopa-loaded nanoparticles (Figure 13) while SEM displayed the hollow spherical nanocapsules (Figure 14).

### 2.5. Drug Loading Efficiency of Poly-Lipo Nanoparticles

The drug loading efficiency was found to be 93%. The poly-lipo nanoparticles had a high drug entrapment efficiency of 85% and though the fabrication was stepwise there was no washing, centrifuging or decanting. It is envisaged that drug incorporation into the nanoparticles is a combination of encapsulation and surface adsorption.

### 2.6. Direct Compression of Poly-Lipo Nanoparticles into Tablet Matrices

All formulations of l-dopa-loaded nanoparticles were directly compressible. Direct compression is indeed a pharmaceutical technique employed for moisture-sensitive drugs such as l-dopa. Moisture, being one of the major causes of instability of drugs, is not utilized in direct compression, unlike wet granulation. The compressibility of the nanoparticles demonstrated the inherent compaction of the polymers which was not eliminated by the process of fabrication of the nanoparticles.

### 2.7. In vitro Drug Release Studies

**Figure 15:**
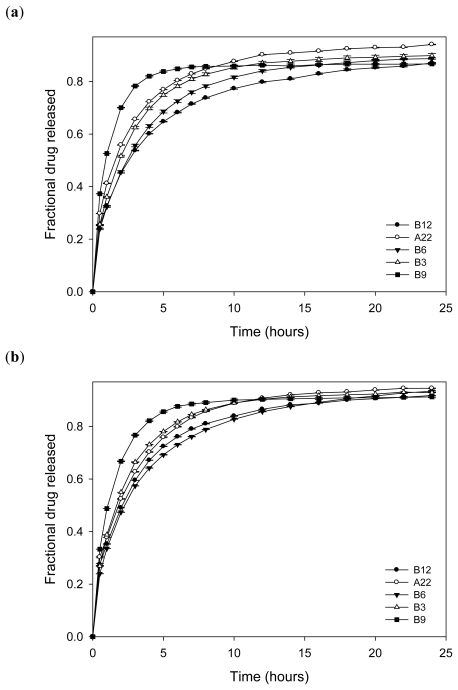
(**a**) Drug release profiles of L-dopa-loaded nanoparticles employing dialysis technique in pH 1.5 buffer; and (**b**) drug release profiles of L-dopa-loaded nanoparticles employing dialysis technique in pH 4.5 buffer.

**Figure 16:**
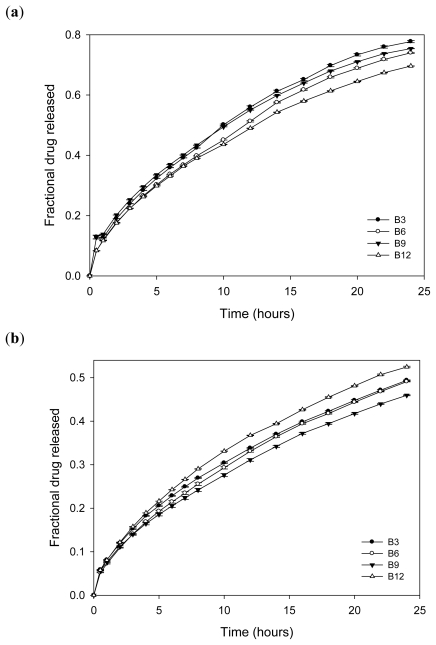
(**a**) Drug release profiles of L-dopa-loaded nanoparticles incorporated into an interpolymeric blend in pH 1.5 buffer; and (**b**) drug release profiles of L-dopa-loaded nanoparticles incorporated into an interpolymeric blend in pH 4.5 buffer.

By the 5th hour, nanosuspensions had released more than 60% of l-dopa while all except B12 and B6 released more than 50% within two hours (Figure 15). Hence Levodopa-loaded nanosuspension can be employed for rapid relief of parkinsonian symptoms. The nanoparticles are anticipated to adhere to the mucosal walls by electrostatic interactions thereby improving the bioavailability of l-dopa. l-dopa release from nanosuspension into the two buffers utilized (pH 1.5 and 4.5) did not differ significantly as shown in Figures 15a and 15b. By the 24th hour, 86–94% of l-dopa was released. However, the release profiles in buffers differed upon incorporating the nanoparticles into an interpolymeric blend (Figures 16a,b) which may be due to the pH responsive nature of the interpolymeric blend. As pH increased, the rate of release of l-dopa decreased. Furthermore, the fractional drug released by 24th hour from the interpolymeric blend was less when compared to nanosuspension due to increased barrier and subsequent diffusion distance. While 78% drug release was the highest released in buffer pH 1.5 by one of the nanoparticulate compositions, 46% was the lowest released in buffer pH 4.5 by the 24th hour.

**Figure 17:**
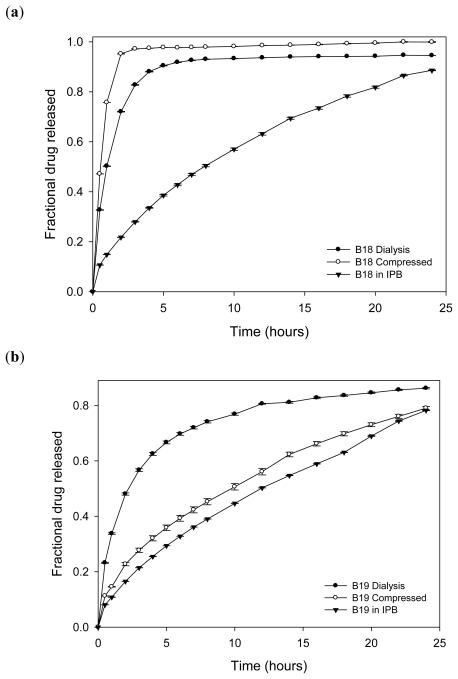
(**a**) Comparative drug release profiles of the different formulations of levodopa-loaded methacrylate copolymer nanoparticles (B18) in pH 1.5; and (**b**) comparative drug release profiles of the different formulations of levodopa-loaded methacrylate copolymer/chitosan nanoparticles (B19) in pH 1.5.

Comparing drug release profiles from the three formulations–nanosuspension, compressed nanoparticles and nanoparticles compressed within an interpolymeric blend, it was observed that incorporating methacrylate copolymer nanoparticles into an interpolymeric blend produced a sustained release of L-dopa as compared to direct compression of the nanoparticles alone (Figure 17a). Since buffer pH 1.5 percolates the compressed methacrylate copolymer nanoparticles, the particles are dispersed in the buffer such that 75% of levodopa is released into the medium at the 1st hour. On the other hand, directly compressed l-dopa-loaded methacrylate copolymer/chitosan nanoparticles remained basically intact with swelling and slight erosion over 24 h with release of l-dopa almost comparable to the release profile of the nanoparticles incorporated into an interpolymeric blend (Figure 17b).

### 2.8. Polymeric Nanoparticles Improve Mechanical Strength

**Figure 18:**
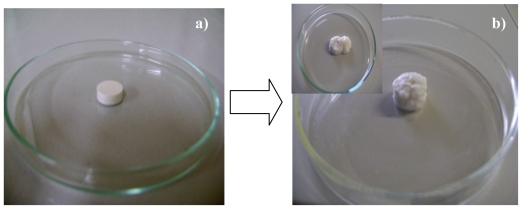
(**a**) Interpolymeric tablet matrix loses; and (**b**) its three-dimensional shape as the pH increases to 4.5 after dissolution studies.

**Figure 19:**
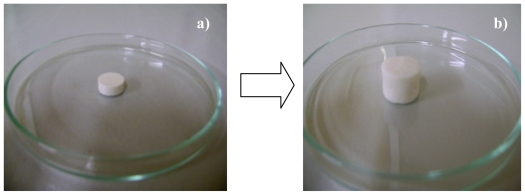
(**a**) interpolymeric tablet matrix shape retained; and (**b**) its three-dimensional shape in pH 4.5 when polymeric nanoparticles are incorporated into it.

The interpolymeric blend being pH responsive maintained its three-dimensional network in pH 1.5 but underwent surface erosion in higher pH such as 4.5. However, in the presence of incorporated nanoparticles, the three-dimensional network was maintained in both buffer types over the 24 h drug release studies. The mechanical strength of the interpolymeric blend was improved in pH 4.5 by the electrostatic interactions between the nanoparticles and the interpolymeric blend. Studies have shown that nanoparticles can be employed to improve the mechanical strength of matrices [69–75]. These studies utilized inorganic nanoparticles to improve mechanical properties. However, in this study polymeric nanoparticles improved the mechanical strength of a polymeric matrix preventing the polymeric matrix’s erosional response at a higher pH. The pictorial diagram of the impact of nanoparticles on the mechanical strength of the interpolymeric blend matrix is shown in Figures 18 and 19.

### 2.9. Magnetic Resonance Imaging

**Figure 20:**
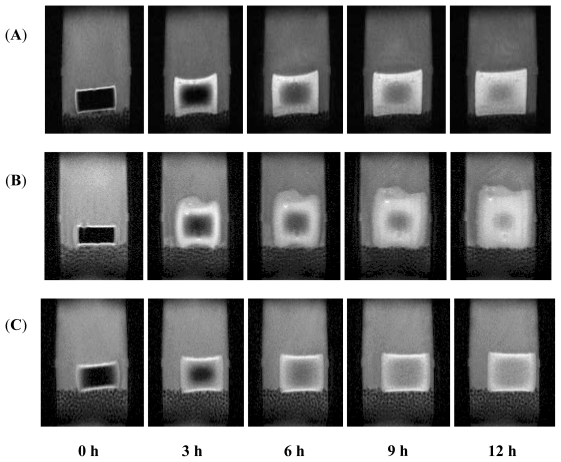
Magnetic resonance images of the mechanical behavioral changes of matrices in different pHs: (**A**) nanoparticles incorporated into interpolymeric blend at pH 1.5; (**B**) interpolymeric blend matrix without nanoparticles at pH 4.5 (**C**) nanoparticles incorporated into interpolymeric blend at pH 4.5 at 0, 3, 6, 9 and 12 h.

Magnetic resonance imaging (MRI) was also employed to observe and confirm the mechanical behaviors that occurred during dissolution studies. Figure 20A displays the images obtained at pH 1.5 upon incorporating the nanoparticles into the interpolymeric blend. Figure 20B shows interpolymeric blend without nanoparticles at pH 4.5 while Figure 20C shows the mechanical behavior of the matrix upon incorporating the nanoparticles into the interpolymeric blend at pH 4.5. The images shown were obtained at zero, 3, 6, 9 and 12 h. The grey part surrounding the matrix is the dissolution medium (buffer). The black part within the matrix is the non-hydrated and non-gelled part of the tablet. As the matrix hydrated, it swelled and gelled, which is shown by the white part, and its thickness increased over time until the matrix was fully hydrated and gelled. As shown, the matrix is a hydrogel which retains its three-dimensional network (shape) at pH 1.5. Figure 20B confirmed the gradual surface erosion that occurs at pH 4.5 without the nanoparticles. The matrix loses its shape at pH 4.5 as it hydrates, erodes and gels. Rapid erosion was not observed because three polymers were employed to prepare the interpolymeric blend with two of them forming interpolyelectrolyte complex. It is typical for hydrogel matrices produced with high-molecular weight polymers not to exhibit rapid erosion as their helical chains form rigid interactions within the polymer molecules [76]. Usually it requires more than 24 h for the interpolymeric blend to erode completely. However, when nanoparticles were added, at pH 4.5 the matrix retained its shape with less penetration of solvent into the matrix as the thickness of the white part was less when compared to images in Figure 20A and consequently less swelling. Hence, magnetic resonance imaging confirmed the mechanical influence of nanoparticles on polymeric matrices.

### 2.10. Molecular Mechanics Assisted Model Building and Energy Refinements

A molecular mechanics conformational searching procedure was employed to acquire the data employed in the statistical mechanics analysis, and to obtain differential binding energies of a Polak–Ribiere algorithm and to potentially permit application to PR and TPP-mediated crosslinking of E100/CHT polymer composite assemblies. MM+ is a HyperChem modification and extension of Norman Allinger’s Molecular Mechanics program MM2 [77], whereas AMBER, is a package of computer programs for applying molecular mechanics, normal mode analysis, molecular dynamics and free energy calculations to simulate the structural and energetic properties of molecules [78].

#### 2.10.1. Molecular Mechanics Energy Relationship (MMER) Analysis

Molecular mechanics energy relationship (MMER), a method for analytico-methematical representation of potential energy surfaces, was used to provide information about the contributions of valence terms, noncovalent Coulombic terms, and noncovalent van der Waals interactions for the crosslinked-polymer morphologies. The MMER model for the potential/steric energy factors in various molecular complexes can be written as:

(1) $$E_{\mathrm{molecule}/\mathrm{complex}}=V_{\Sigma }=V_{b}+V_{\theta }+V_{\phi }+V_{ij}+V_{hb}+V_{el}$$

(2) $$E_{\mathrm{CHT}}=35.555V_{\Sigma }=3.120V_{b}+18.035V_{\theta }+25.774V_{\phi }+13.323V_{ij}-24.697V_{el}$$

(3) $$E_{\mathrm{PR}}=6.717V_{\Sigma }=0.145V_{b}+2.477V_{\theta }+4.743V_{\phi }-0.484V_{ij}-0.163V_{hb}$$

(4) $$E_{\mathrm{CHT}-\mathrm{PR}}=22.230V_{\Sigma }=12.683V_{b}+1.875V_{\theta }+27.680V_{\phi }+7.176V_{ij}-0.596V_{hb}-26.757V_{el}[\Delta E_{\mathrm{BINDING}}=-33.476\;\mathrm{kcal}/\mathrm{mol}]$$

(5) $$E_{E100}=100.577V_{\Sigma }=10.018V_{b}+45.058V_{\theta }+13.454V_{\phi }+32.052V_{ij}-0.0068V_{hb}$$

(6) $$E_{E100-\mathrm{PR}}=89.724V_{\Sigma }=10.579V_{b}+57.476V_{\theta }+39.343V_{\phi }-16.028V_{ij}-1.647V_{hb}[\Delta E_{\mathrm{BINDING}}=-24.287\;\mathrm{kcal}/\mathrm{mol}]$$

(7) $$E_{E100-\mathrm{PR}-\mathrm{CHT}}=39.230V_{\Sigma }=13.645V_{b}+89.514V_{\theta }+91.042V_{\phi }-122.797V_{ij}-2.847V_{hb}-29.327V_{el}[\Delta E_{\mathrm{BINDING}}=-150.638\;\mathrm{kcal}/\mathrm{mol}]$$

(8) $$E_{\mathrm{TPP}}=199.744V_{\Sigma }=1.927V_{b}+93.088V_{\theta }+1.599V_{\phi }+0.046V_{ij}+103.082V_{el}$$

(9) $$E_{\mathrm{CHT}-\mathrm{TPP}}=901.408V_{\Sigma }=18.542V_{b}+514.621V_{\theta }+54.501V_{\phi }+28.920V_{ij}-1.065V_{hb}+285.889V_{el}[\Delta E_{\mathrm{BINDING}}=-132.867\;\mathrm{kcal}/\mathrm{mol}]$$

where, *V*_∑_ is related to total steric energy for an optimized structure, *V*_b_ corresponds to bond stretching contributions (reference values were assigned to all of a structure’s bond lengths), *V*_θ_ denotes bond angle contributions (reference values were assigned to all of a structure’s bond angles), *V*_ϕ_ represents torsional contribution arising from deviations from optimum dihedral angles, *V*_ij_ incorporates van der Waals interactions due to non-bonded interatomic distances, *V*_hb_ symbolizes hydrogen-bond energy function and *V*_el_ stands for electrostatic energy.

#### 2.10.2. Energy-Minimizations Involving Crosslinked-Polymer Morphologies

**Figure 21:**
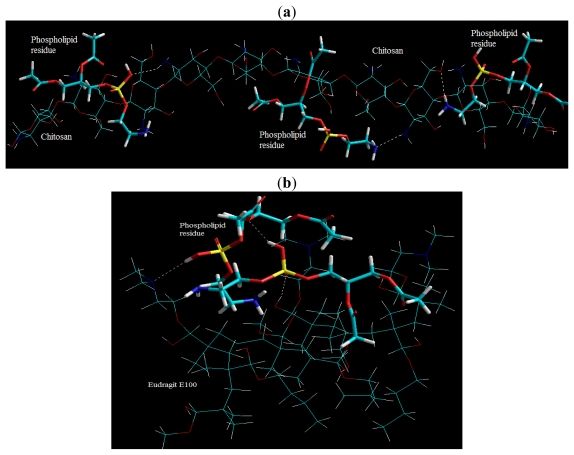
Visualization of geometrical preference of Phospholipid residues in complexation with: (**a**) Chitosan; and (**b**) Eudragit E100 after molecular mechanics simulations. The atoms forming the hydrogen bonds are emphasized by dotted lines after recomputing the H bonds after energy minimizations. Color codes: C (cyan), O (red), N (blue), P (yellow) and H (white).

The energy changes brought about by crosslinking of E100 and Chitosan with PR are given in equations 2 to 6 and the resulting geometrical minimizations are depicted in Figure 21. The total change in energy is calculated as the sum of an internal energy component, calculated from bond stretching, angle bending, and torsional rotation, and a non-bonded component calculated from coulombic and van der Waals interactions. It is evident from Figure 21a that PR may crosslink CHT by forming hydrogen bonds between their functional groups such as PO_4_ ^−^… NH, NH… OH^−^ and NH… NH crosslinking. The Δ*E*_BINDING_ = −33.476 kcal/mol proved that CHT-PR forms a stable structure stabilized by all the three non-bondng interactions viz., van der Waals forces, hydrogen bonding and electrostatic interactions with London dispersion forces playing the major part (Equations 4–6). These interactions confirmed the previously reported interactions between chitosan and phospholipids (lecithin) as discussed earlier [63–68].

**Figure 22:**
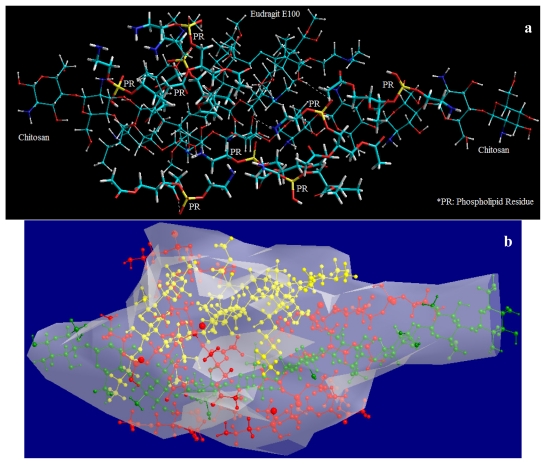

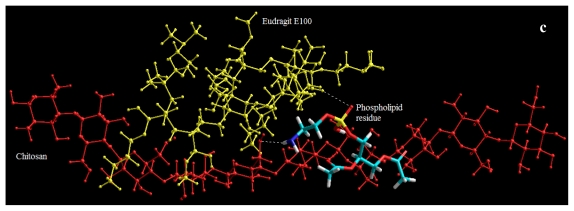
Visualization of geometrical preference of Chitosan and Eudragit in complexation with Phospholipid residues (PR) after molecular mechanics simulations: (**a**) E100-PR-CHT molecular complex in full geometry; (**b**) Connolly molecular electrostatic potential surfaces in transparebt display mode showcasing the nanoparticlulate system; and (**c**) A typical conformation showcasing the bridging of E100 and CHT by phospholipid residue. Color codes: C (cyan), O (red), N (blue), P (yellow) and H (white).

**Figure 23:**
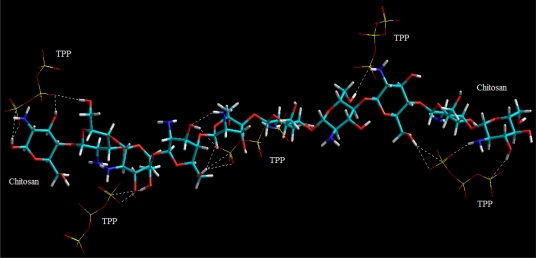
Visualization of geometrical preferences of TPP (stick rendering) in complexation with CHT (tube rendering) after molecular mechanics simulations. Color codes: C (cyan), O (red), N (blue), P (yellow) and H (white).

It is apparent form Figure 21b that E100 can also be crosslinked by PR, as postulated earlier in the paper, forming both intra- and inter-molecular bonding. Figure 21b depicts that the complexing of phosphate with amine group proceeds via formation of both the hydrogen bond NH... O=P and CO…O=P and also the N^δ−^...p^δ+^ bond [79]. The formation of stabilized structure of E100-PR with Δ*E*_BINDING_ = −24.287 kcal/mol also confirms the crosslinking action of TPP. The main difference between CHT-PR and E100-PR crosslinking is that there is no electrostatic interaction involved between E100 and PR. E100-PR is thus stabilized mainly by van der Waals hydrophobic interaction and negligibly by H-bonding (Equations 5 and 6). The above crosslinking reactions are formed the basis of our postulation, which states that the incorporation of lecithin might act as a bridge for interpolymeric crosslinking. To elucidate this concept, we modeled E100 and CHT together with PR (E100-PR-CHT), molecularly dispersed within the polymeric matrix (Figure 22). After global energy and geometry minimization, we observed that it is possible for PR to form an interpolymeric bridge between E100 and CHT as depicted in Figure 23c. Surprisingly, the ΔE_BINDING_ for the trimolecular complex was −150.638 kcal/mol, which was ~5 times more stabilized than the individual polymer complex of E100-PR or CHT-PR (Equation 7).

The final conformation model of E100-PR-CHT molecular network was generated for formable complex structures in relation to the cooperative ion-pair binding of the carbonyl (−CO), hydroxyl (−OH^−^), protonated amine (−NH_3_ ^+^) groups of the polymers with the quaternary phosphonium ion (PO_4_ ^−^) and protonated amine (−NH_3_ ^+^) groups of the crosslinker (Figure 21a). The strong binding affinity in E100-PR-CHT was due to the hydrophobic interactions and charge distribution caused by the interacting moieties which act in co-operation with the short range van der Waal attractions and secondary interactions such as hydrogen bonding (Equation 7). Interestingly, the stabilized van der Waals energy demonstrated the importance of a structural backbone fit between the host and guest molecule. Figure 21b depicts the energy-minimized van der Waal radii structure of E100-PR-CHT where, due to the flexibility of the polymer chains, the relevant segments of polymers arrange their configuration to form a remarkable structure fit between the various functional groups, the electrostatic, van der Waals and H-bond interactions being almost optimized.

Apart from these, the chitosan polymeric matrix in particular was further stabilized and crosslinked due to the addition of sodium tripolyphosphate (TPP) as shown in Figure 23 and Equations 8 and 9. The CHT-TPP molecular complex demonstrated a Δ*E*_BINDING_ of −132.867 kcal/mol. The complex is mainly stabilized by nonbonding interactions in terms of London dispersion forces, H-H bonding and ion pair-ion pair electrostatic interactions (Equations 8 and 9). However, it is apparent in Figure 23 that direct linking of adjacent glucosamine units may have brought about the largest change in energy relative to uncrosslinked states. The following possible crosslinks were formed: PO_4_ ^−^-OH crosslinking, PO_4_^−^-NH crosslinking, OH-PO_4_^−^-OH crosslinking, NH-PO_4_^−^-NH crosslinking and OH-PO_4_^−^-NH crosslinking.

Although the complex morphologies were finally stabilized by dominating non-bonding interactions, the chain interactions culminating from the crosslinking agents such as Lecithin (a phospholipid) and sodium tripolyphosphate (conjugate base of triphosphoric acid) may cause disturbance at high crosslink densities resulting in distortion of bond lengths, angles, and torsions from their equilibrium values eventually causing strained-unstable-rigid-framework (Figure 22b). Additionally, the intermolecular crosslinking may initiate a significant axial stress due to buildup of the adjacent crosslinks. This provides a reasonable explanation for the experimentally observed controlled release behavior of the nanoparticle formulations due to formation of a dense polymeric matrix owing to this very crosslinking mechanism of PR and TPP.

These modeling results are in corroboration with the observed experimental results where it was postulated that the color change after the addition of crosslinking agent may be due to energy perturbation. Additionally, free flowing particles generated after the inclusion of TPP may be further attributed to less space in the particles’ matrices for solvent and water molecules due to the matrix stability and robustness displayed in energy minimized geometrical configurations. Furthermore, this matrix stability and robustness may induce a low degree of matrix loss, minimal swelling and limited distension resulting in a sustained and prolonged release of levodopa from the nanoparticle formulations. The spectral analysis also corroborated with the mechanistic simulation in terms of disappearance of peaks and emergence of new peaks along with band shifting and broadening involved in coupling and complexation (FTIR structural variation analysis) which strengthens the experimental and computational correlation.

## 3. Experimental Section

### 3.1. Materials

Methacrylate copolymer (Eudragit E100) was purchased from Evonik Röhm GmbH & Co. KG (Evonik Röhm GmbH and Co. KG, Darmstadt, Germany), chitosan (food grade), (Wellable group, Fujian, China), sodium tripolyphosphate (TPP) (Sigma-Aldrich, Steinheim, Germany), lecithin from egg yolk (Lipoid E PC S, a gift from Lipoid AG, Ludwigshafen, Germany), 3-(3,4-Dihydroxyphenyl)-l-alanine (levodopa), potassium phosphate monobasic (KH_2_PO_4_) (Sigma-Aldrich Inc., Steinheim, Germany), acetic acid glacial, hydrochloric acid (HCl) (Rochelle Chemicals, Gauteng, South Africa), potassium chloride (KCl) (Saarchem, Krugersdorp, South Africa), ortho-phosphoric acid (BDH Chemicals, Poole, England) and chloroform (Rochelle Chemicals, Gauteng, South Africa). All other reagents used were of analytical grade and were employed as purchased.

### 3.2. Nanofabrication of Polymer-Lipid Nanoparticles

Weighed quantities of methacrylate copolymer and varying quantities of methacrylate copolymer with chitosan were dissolved in 10 mL 0.2N HCl and 100 mg of l-dopa was added into the polymeric solution. Lipoid E PC S (100 mg) was dissolved in 1mL of chloroform and added to levodopa-loaded polymeric solution under mechanical agitation for 10 min. Varying concentrations of TPP dissolved in 0.2 N acetic acid were added under agitation for another 10 min, pre-freezed at − 70 °C for 24 h and thereafter lyophilized for 48 h. The different formulations of nanoparticles are shown in Table 3.

### 3.3. Determination of pH and Absorbance Changes During Fabrication

Initial pH values for the polymer solutions were obtained followed by changes in pH during the addition of the polymer solutions (in a stepwise manner) to the acidic media. Hence, four pH values were obtained at the end of fabrication. The changes in absorbance from the polymer solution, on addition of lecithin and TPP afterwards were obtained. The absorbance changes were obtained in the absence of Levodopa (l-dopa)

### 3.4. Computational Modeling of the Fabrication of Multi-Crosslinked Nanoparticles

Computational modeling was performed to expound the interactions between the polymers and the crosslinking agents as well as the mechanism of the formation of nanoparticles. Models and graphics depicting the mechanisms of interactions were generated on ACD/l-Lab, V5.11 (Add-on) software (Advanced Chemistry Development Inc., Toronto, Canada, 2000). Interactions were interpreted by employing some general chemistry concepts and chemometric modeling concepts.

### 3.5. Size and Surface Charge Analyses of the Poly-Lipo Nanoparticles

Nanoparticle size, size distribution as well as zeta potential analyses were carried out using ZetaSizer NanoZS (Malvern Instruments, Malvern, UK) instrument equipped with non-invasive backscatter technology set at an angle of 173°. The nanoparticles sizes and zeta potentials were profiled following the addition of lecithin, then after addition of TPP and finally after lyophilization.

### 3.6. Fourier Transform Infra-Red (FTIR) Spectroscopy of the Poly-Lipo Nanoparticles

FTIR spectra over the range of 4000–650 cm^−1^ were obtained for the native polymers employed and the poly-lipo nanoparticles using a PerkinElmer spectrometer (PerkinElmer Spectrum 100, Beaconsfield, United Kingdom) to elucidate the chemical structural transitions that occurred during nanofabrication.

### 3.7. Microscopical Analysis of the Levodopa-Loaded Poly-Lipo Nanoparticles

The surface morphology analyses of the poly-lipo nanoparticles were undertaken by performing digital microscopy, transmission electron microscopy (TEM) and scanning electron microscopy (SEM). The digital microscopical images of the poly-lipo nanoparticles after synthesis were obtained using Olympus digital microscope; Olympus SZX-ILLD2-200 (Olympus Corporation, Tokyo, Japan). The nanosuspension was diluted tenfold with deionized water and drops were deposited on formvar coated nickel grids. They were allowed to dry in sealed petri dish and later viewed under TEM (JEOL-JEM 100S transmission electron microscope, Tokyo, Japan). Furthermore, lyophilized nanoparticles were thinly spread on a carbon tape, coated with gold-palladium and viewed under SEM (JEOL-JEM 840 scanning electron microsope, Tokyo, Japan) at a voltage of 15 keV and current 6 × 10^−10^ Amp.

### 3.8. Determination of Drug Loading and Drug Entrapment Efficiency of the Poly-Lipo Nanoparticles

Percentage drug loading efficiency was determined gravimetrically to assess the capacity of the nanoparticles with regards to the amount of the loaded drug in the nanoparticles. The percentage drug loading was calculated based on the weights of the incorporated drug and the nanoparticles employing Equation 10:

(10) $$\text{Drug Loading }(\%)=\frac{\text{Amount of drug in nanoparticles}}{\text{Amount of nanoparticles}}\times 100$$

The drug entrapment efficiency was determined by dispersing the poly-lipo nanoparticles in 0.1N HCl and the amount of the drug in the medium was assessed spectrophotometrically to obtain the amount of drug in the poly-lipo nanoparticles with respect to the amount of drug used in the fabrication, employing Equation 11:

(11) $$\text{Drug entrapment efficiency }(\%)=\frac{\text{Actual Amount of drug}}{\text{Theoretical amount of drug}}\times 100$$

### 3.9. Direct Compression of the Poly-Lipo Nanoparticles

#### 3.9.1. Exclusive Direct Compression of the Poly-Lipo Nanoparticles

The lyophilized poly-lipo nanoparticles were directly compressed without additives or excipients in order to elucidate their drug release profiles unaided. The compression was undertaken employing a Carver hydraulic press (Carver Industries Inc., Wabash, IN, USA) at 3 tons.

#### 3.9.2. Incorporation of Poly-Lipo Nanoparticles into an Interpolymeric Blend

The incorporation of poly-lipo nanoparticles into the interpolymeric blend was undertaken by employing an interpolymeric blend (IPB) comprised of methacrylate copolymer, carboxymethyl cellulose and locust bean. The L-dopa loaded poly-lipo nanoparticles were blended with the interpolymeric blend without adding any other excipients or additives. The nanoparticles-loaded interpolymeric blend was directly compressed employing Carver hydraulic press (Carver Industries Inc., Wabash, IN, USA) at 3 tons. The composition of the nanoparticles utilized for incorporation into the interpolymeric blend is shown under Section 3.2, Table 3 (S/N 1-7).

### 3.10. *In vitro* Drug Release Studies

Drug release was assessed using USP apparatus II dissolution system (Erweka DT 700, Erweka GmbH, Heusenstamm, Germany). Temperature and stirring rate were 37 ± 0.5 °C and 50 rpm respectively while the dissolution media were 900 mL buffer pH 1.5 (standard buffer KCl/HCl) and pH 4.5 (0.025 M KH_2_PO_4_/H_2_PO_4_). Samples were withdrawn at intervals and replaced with the same volume of fresh medium to maintain sink conditions. The amount of L-dopa released was quantified using UV spectrophotometer (LAMBDA 25 UV/Vis spectrophotometer, PerkinElmer, MA, USA). The drug release profiles were obtained from three formulations of L-dopa-loaded nanoparticles.

#### 3.10.1. Drug Release Studies of Nanosuspension Employing Dialysis Technique

Various quantities (depending on the varying composition) of l-dopa-loaded nanoparticles dispersed in 10mL of dissolution media; buffer pH 1.5 (standard buffer KCl/HCl) and pH 4.5 (0.025 M KH_2_PO_4_/H_2_PO_4_) at separate periods were introduced into dialysis tubing (Dialysis tubing cellulose membrane, Ave flat width–33 mm, diameter–21 mm when full, M.W. 12,400, Sigma Aldrich, Steinheim, Germany). Both ends of the tubing were tightly sealed and placed in a dissolution vessel with 900 mL of buffer and stainless ring-mesh assemblies were employed to keep the tubings in place to prevent erratic floatation due to unstable hydrodynamics above the paddle. Before incorporation of nanoparticles into the tubing, the dialysis tubing was immersed in deionized water for 3 h to remove glycerol and sulfide from the tubing. The drug release studies were conducted over 24 h.

#### 3.10.2. Drug Release Studies of the Compressed Matrices

The second and third drug release studies involved the matrices of the L-dopa-loaded nanoparticles alone and the L-dopa-loaded nanoparticles incorporated into an interpolymeric blend in pH 1.5 buffer (standard buffer KCl/HCl) and pH 4.5 (0.025 M KH_2_PO_4_/H_2_PO_4_) over 24 h. The apparatus and parameters employed for dissolution are as stated above.

### 3.11. Magnetic Resonance Imaging of Mechanical Behavior

A magnetic resonance system with digital MARAN-i System configured with a DRX2 HF Spectrometer console (Oxford Instruments Magnetic Resonance, Oxon, UK) equipped with a compact 0.5 Tesla permanent magnet stabilized at 37 °C and a dissolution flow through cell was employed for the viewing of the mechanical behaviors of the matrices. After duly configuring, optimizing the shims and probe tuning, the cone-like lower part of the cell was filled with glass beads to provide laminar flow at 16 mL/min of the solvents employed. The matrices were placed in position each time within the cell which in turn was positioned in a magnetic bore of the system and magnetic resonance images were acquired hourly over 12 h with MARAN-i version 1.0 software. The image was acquired after setting the frequency offset and testing gain employing RINMR version 5.7 under continuous solvent flow conditions. MARAN-i software comprises image acquisition software and image analysis software. The image acquisition parameters are depicted in Table 4.

### 3.12. Static Lattice Atomistic Simulations

Molecular Mechanics Computations in vacuum, which included the model building of the energy-minimized structures of multi-polymer-crosslinker complexes, were performed using the HyperChem^TM^ 8.0.8 Molecular Modeling System (Hypercube Inc., Gainesville, FL, USA) and ChemBio3D Ultra 11.0 (CambridgeSoft Corporation, Cambridge, UK) [80]. The structures of Eudragit (E100-four monomer units), Phospholipid residues (PR) and TPP were generated as a 3D model from standard bond lengths and angles employing ChemBio3D Ultra whereas the structure of chitosan (CHT-ten glucosamine oligosaccharide units) was generated using sugar builder module on HyperChem 8.0.8. The generation of the overall steric energy associated with the energy-minimized structures was initially executed via energy-minimization using MM+ force field and the resulting structures were again energy-minimized using the Amber 3 (Assisted Model Building and Energy Refinements) force field. The conformer having the lowest energy was used to create the polymer-crosslinker complexes. A complex of one molecule with another was assembled by disposing them in a parallel way, and the same procedure of energy-minimization was repeated to generate the final models: CHT-TPP, CHT-PR, E100-PR and E100-PR-CHT. Full geometry optimizations were carried out in vacuum employing the Polak–Ribiere conjugate gradient method until an RMS gradient of 0.001 kcal/mol was reached. Force field options in the AMBER (with all hydrogen atoms explicitly included) and MM+ (extended to incorporate non-bonded cut-offs and restraints) methods were the HyperChem 8.0.8 defaults. For calculations of energy attributes, the force fields were utilized with a distance-dependent dielectric constant scaled by a factor of 1. The 1–4 scale factors are following: electrostatic 0.5 and van der Waals 0.5 [80].

## 4. Conclusions

This study demonstrates the feasibility of fabricating of L-dopa-loaded nanoparticles. The obtained nanoparticles were hollow, capsular, nanoparticulate complex with 93% drug loading efficiency. The desired rate of drug release from methacrylate copolymer can be modulated by formulating levodopa-loaded nanoparticles as a suspension, then directly compressing the nanoparticles into a tablet matrix or by loading the nanoparticles into a polymeric blended tablet matrix. Twenty-four hour sustained release of levodopa from methacrylate copolymer nanoparticles was achieved by embedding the nanoparticles within an interpolymeric blended tablet matrix while sustained drug release from methacrylate copolymer/chitosan nanoparticles can be achieved by directly compressing the nanoparticles into a tablet matrix. This study also elucidates the potentials of polymeric nanoparticles to enhance the mechanical strength of polymeric matrices. Furthermore, experimental and theoretical investigations on the effect of crosslinking on the release of L-dopa from the nanoparticles formulations were in excellent agreement. The experimentally observable drug release could be predicted by the computational method and the energy calculations allowed for a prediction of the interaction mechanisms. The developed delivery system may find applications in oral, sustained and localized drug delivery.

## Figures and Tables

**Table 1:** Comparative pH changes during nano-fabrication.

Formulation Code	Polymer Solution	Addition of L-dopa	Polymer + L-Dopa + Lecithin	Polymer + L-dopa + Lecithin + TPP
A22	1.17	1.31	1.36	3.15
B3	1.17	1.34	1.40	1.73
B6	1.18	1.36	1.41	1.78
B9	1.13	1.28	1.28	1.68
B12	1.14	1.19	1.23	1.78
B13	1.14	1.19	1.23	1.78
B18	1.13	1.28	1.28	2.37
B19	1.18	1.36	1.41	2.82

pH of 0.2N HCL was 1.00.

**Table 2:** Changes in absorbances during nano-fabrication.

Polymer Composition	Polymer Solution	Addition of Lecithin	Addition of TPP
EE100	0.0135	0.5681	0.4876
Chitosan	0.1382	3.3501	3.5597
EE100 + Chitosan	0.0589	2.7885	3.1930

EE100-methacrylate copolymer.

**Table 3:** Components and the respective quantities employed for nanoparticles formation.

S/N	Formulation Code	Eudragit (mg)	Chitosan (mg)	L-dopa (mg)	Lecithin (mL)	TPP (mg)
1	A22	150	150	100	1.00	250
2	B3	150	50	100	1.00	50
3	B6	100	100	100	1.00	50
4	B9	200	–	100	1.00	50
5	B12	50	50	100	1.00	100
6	B18	200	–	100	1.00	150
7	B19	100	100	100	1.00	150
8	C0	–	200	100	1.00	150
9	C1	–	200	100	1.00	–
10	B18_0_	200	–	100	1.00	–
11	B19_0_	100	100	100	1.00	–

**Table 4:** Image acquisition parameters applied during magnetic resonance imaging using MARAN-i.

S. No.	Parameter	Value
**1.**	Imaging protocol	FSHEF
**2.**	Requested gain (%)	1.90
**3.**	Signal strength	71.62
**4.**	Average	2
**5.**	Matrix size	128
**6.**	Repetition time (ms)	1000.00
**7.**	Spin Echo Tau (ms)	6.00
**8.**	Image acquired after	60 min
**9.**	Total scans	64
